# The contact lens dry eyes questionnaire (CLDEQ-8) validation and ocular surface dysfunction among soft contact lens wearers

**DOI:** 10.5935/0004-2749.20220015

**Published:** 2022

**Authors:** Marcelo Ribeiro, Matheus Schmitz Vieira, Gabriel Gorgone, Lucas Yunes Cominatto Barbosa, Alexandre Ricardo Abdel Fattah Martini, Milton Agrizzi David, Denise Oliveira Fornazari, Monica Alves, Carlos Eduardo Leite Arieta

**Affiliations:** 1 Department of Ophthalmology and Otorhinolaryngology, Faculty of Medical Sciences, Universidade Estadual de Campinas, Campinas, SP, Brazil

**Keywords:** Contact lens, Dry eye syndrome, Ocular surface, Survey and questionnaire, Reproducibility of results, Lente de contato, Síndrome do olho seco, Superfície ocular, Inquérito e questionário, Reprodutibilidade de resultados

## Abstract

**Purpose:**

To translate and validate the Contact Lens Dry Eyes Questionnaire (CLDEQ-8) to Portuguese language and to describe the impact of soft contact lenses on the ocular surface.

**Methods:**

We conducted a descriptive transversal study with the aim to: (1) translate and validate the CLDEQ-8 questionnaire to Portuguese language and (2) apply the CLDEQ-8 to a group of contact lens wearers along with a broad evaluation of the impact of soft contact lens on the ocular surface. The evaluation of the impact of soft contact lens was performed for a study population of 81 subjects, categorized in two groups: Group A: 61 contact lens wearers and Group B (control): 20 noncontact lens wearers. The study exclusion criteria were rigid contact lens wear, systemic or ocular diseases, the use of medications predisposing to ocular surface damage, and previous ocular surgeries.

**Results:**

For the CLDEQ-8 questionnaire translation and validation, Kappa agreement values were ≥0.7 in all questions, implying a good agreement between the Portuguese and English language versions. Considering the ocular surface evaluation of the subjects, all parameters differed in Soft contact lens wearers when compared with the controls (p<0.05), except in those related to tear volume, such as the tear meniscus height and Schirmer test.

**Conclusions:**

This study provided a translated and validated Portuguese version of CLDEQ-8 questionnaire, which represents an important tool for the evolution of contact lens wearers. The broad evaluation of the ocular surface revealed an association between soft contact lens wearing and ocular surface disturbances.

## Abstract (pt)

**Objetivo:**

Traduzir e validar o questionário de olho seco e lentes de contato (CLDEQ-8) para o português e descrever o impacto das lentes de contato gelatinosas na superfície ocular.

**Métodos:**

Estudo transversal e descritivo com o objetivo de (1) traduzir e validar o CLDEQ-8 para o português e (2) aplicar o CLDEQ-8 em um grupo de usuários de lentes de contato, juntamente com uma ampla avaliação do impacto das lentes gelatinosas na superfície ocular. A avaliação do impacto das lentes gelatinosas foi realizada em uma amostra composta por 81 indivíduos, divididos em dois grupos: 61 usuários de lente de contato (Grupo A) e um grupo controle de 20 não usuários (Grupo B). Como critério de exclusão: usuário de lentes de contato rígidas, doenças sistêmicas ou oculares prévias, uso de medicamentos que podem causar danos a superfície ocular e cirurgias oculares prévias.

**Resultados:**

Para a tradução e validação do questionário CLDEQ-8, os valores de concordância Kappa foram iguais ou superiores a 0,7 em todas as perguntas, o que implica em uma boa concordância entre as versões em português e inglês. Considerando a avaliação da superfície ocular dos sujeitos, todos os parâmetros diferiram nos usuários de lente de contato em comparação com os controles (com p<0,05), exceto naqueles relacionados ao volume lacrimal, como altura do menisco lacrimal e teste de Schirmer.

**Conclusões:**

Este estudo forneceu uma versão traduzida para o português e validada do questionário CLDEQ-8, que representa uma importante ferramenta na avaliação de usuários de lente de contato. A avaliação da superfície ocular realizada demonstra a relação entre o uso de lentes de contato gelatinosas e os distúrbios da superfície ocular.

## INTRODUCTION

Soft contact lens (SCL) has been popular since its introduction to the market in 1970, a decade after the Food and Drug Administration approval, although discomfort and dry eye have been some of the major issues, reported in approximately 50% of the wearers, with a literature statistics of 5%-94%^(1-3)^. These issues present as acute or chronic ocular symptoms, with or without visual disturbances, and are mostly related to the disturbances of the ocular surface that interrupt the contact lens wearing experience^(4)^. Different interactions among SCL, cornea, conjunctiva, and meibomian glands are trigged by friction to cause tear in the film and thereby ocular surface dysfunction^(5-7)^. The exact mechanisms and the range of clinical presentation in this situation, however, remains unknown^(3)^. Dry eye is another important contributing factor to discomfort, which is more common in contact lens wearers and the main reason for discontinuation of SCL wear^(8)^. Dry eye has been diagnosed by clinical signs such as conjunctival hyperemia, ocular surface staining, and ocular symptoms, but the correlation between these clinical signs and symptoms have not been clearly established yet.

Several factors may indicate discomfort in SCL wear, such as the type of material, design, adaptation, wear schedule, contact lens care, ocular surface condition (dry eye), environment exposure (e.g., to factors such as humidity, wind, and temperature), occupation (e.g., devices display exposure), drugs, age, and sex^(1)^.

In this context, questionnaires have become useful tools to identify contact lens intolerance, since the detection of dry eye symptoms can be more important than clinical evaluations^(9)^. The Contact Lens Dry Eyes Questionnaire (CLDEQ-8) has been indicated by the Tear Film Ocular Surface Society (TFOS) as the best tool to identify discomfort related to dry eye in contact lens wearers^(10)^. This is an English language questionnaire that was developed in 2012^(11)^, and its use has demanded translation and validation of the original version to other languages, such as Portuguese.

Considering the popularity of SCL wear and the magnitude of discomfort related to the same, we aimed to translate and validate the CLDEQ-8 questionnaire to the Portuguese language and then use it to test its applicability in clinical practice and research as well as to evaluate the impact of SCL wear on the ocular surface. The validated version was applied to a cohort of SCL wearers, followed by a broad evaluation of the ocular surface parameters.

## METHODS

The present study was a descriptive, transversal study conducted in the Department of Ophthalmology at the University of Campinas (UNICAMP) for the following purpose: (1) to translate and validate the CLDEQ-8 questionnaire to the Portuguese language and (2) the application of CLDEQ-8 to a group of contact lens wearers along with a broad evaluation of the impact of SCL on the ocular surface. The study protocol was approved by the Ethics Committee of UNICAMP; the tenets of the Declarations of Helsinki were followed; and the subjects provided their written signed and informed consent forms.

The study population consisted of 81 subjects, who were categorized in two groups: Group A including 61 contact lens wearers and Group B (control) including 20 noncontact lens wearers. Following a sample size number of 20, a proportion of 2:1 was planned. As the invitation to participate in this study was sent across all university social platforms, a large number of contact lens wearers were enrolled and considered in the statistical analysis. To validate the power of our sample, we performed a *post hoc* calculation for each tested variable.

The participants included students and employees of the UNICAMP and of age >18 years. The following were the exclusion criteria for the participants: rigid contact lens wear, systemic or ocular diseases (such as Sjögren Syndrome and pterygium), the use of medications that can predispose to ocular surface damage (e.g., anticholinergics), or previous ocular surgeries (such as refractive surgery or keratoplasty).

To obtain a scientifically accurate translation and transcultural validation of the original English version of the questionnaire into the target Portuguese-language version, we followed a three-phase process after the acceptance from the original authors. First, the initial translation and transcultural adaptation of the English version to the Portuguese language was performed by two independent translators, followed by evaluation by an interdisciplinary panel (constituted by three representatives: two professors of the department and one resident) of the translated version. Second, the Portuguese version was back translated into the English language by two independent native speakers, followed by evaluation and comparison with the original English version by the same interdisciplinary panel. Third, the final version of the questionnaire was applied to a selected population of 30 participants to verify the interand intra-observer concordance.

Contact Lens Dry Eye Questionnaire (CLDEQ-8) is a questionnaire composed of eight questions^(11)^ that was translated and validated to the Portuguese language (Supplementary File 1) for use in the present study, which is a standardized process in literature^(12-14)^ and described below:

1. Two native Portuguese speakers translated the original English language version of the questionnaire to the Portuguese language.
2. An interdisciplinary committee evaluated both the English and Portuguese-language versions to ensure an adequate translation and transcultural adaptation without any alteration that could affect the applicability of the questionnaire.
3. Two native English speakers back translated the questionnaire.
4. The interdisciplinary committee revaluated the back-translated document through comparison with the original version.
5. Two independent observers applied the Portuguese-language questionnaire to a sample of 30 persons at distinct time points. The participants included volunteers from the hospital staff and medical students.
6. A cohort of 30 subjects who responded to the questionnaires applied by both the observers was duly informed about the study goals and their signed informed consent were obtained.
7. Statistical analysis of the responses was performed to determine correlations and Kappa agreement values. Here the minimum and maximum agreement scores were 0 and 1, respectively. Interclass correlation coefficients values were classified as follows: <0.4 bad, 0.4-0.59 moderate, 0.6-0.79 good, and ≥0.8 excellent. The differences were significant at p<0.05. (Figure 1).
  **Figure 1:**
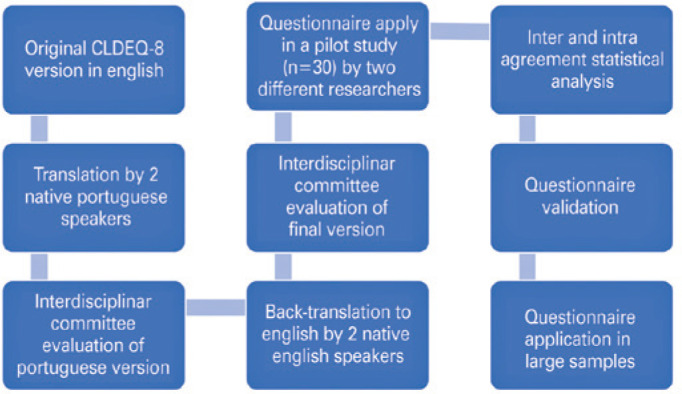
Flowchart of CLDEQ-8 questionnaire translation and validation process.

The validated version of CLDEQ-8 questionnaire was then applied to a group of contact lens wearers. Environmental factors and contact lens habits and ocular surface disease symptoms were investigated by using the Ocular Surface Disease Index (OSDI) questionnaire. A comprehensive set of objective and subjective tests were performed by experienced ophthalmologists to evaluate all aspects of the ocular surface.

The device OCULUS-Keratograph 5M was used to perform non-invasive tear break-up time (NITBUT), conjunctival hyperemia scores, Tear Meniscus Height (TMH), and meibography, in accordance with the manufacturer’s recommendations. Moreover, fluorescein corneal staining (FCS), Schirmer test, lissamine green ocular surface staining (LGOSS) were performed.

The NITBUT, TMH, and conjunctival hyperemia scores were evaluated and classified. The upper and lower eyelids were turned over, and the meibomian glands were documented with a noncontact infrared method by using a Keratograph device. Partial or complete loss of the meibomian glands was scored by using the following grades (meiboscore) for each eyelid: grade 0 (no loss of meibomian glands), grade 1 (the affected area was <25% of the total area occupied by the meibomian glands), grade 2 (the affected area was 25%-50% of the total area occupied by the meibomian glands), grade 3 (the affected area was 50%-75% of the total area occupied by the meibomian glands), and grade 4 (the affected area was >75% of the total area occupied by the meibomian glands)^(15)^.

Ocular surface staining was performed after objective examination. For FCS after 1% fluorescein instillation grades from 0 to 3 were summed from five different zones (totaling 0-15). Corneal fluorescein staining was evaluated by cobalt blue illumination following the 0-15 point NEI/ Industry scale (grades of 0-3 for five regions of the ocular surface: central, nasal, temporal, superior, and inferior), after the TFBUT measurements^(16)^. LGOSS was evaluated in three different zones and over the measurement scale of 0-3 (totaling 0-15). Finally, Schirmer’s test was performed without using any anesthetic drops^(14,17,18)^. Conjunctival staining assessment used a grading scheme as described by van Bijsterveld in accordance with the modified 0-9 point NEI/industry scale, where the grades of 0-3 were assigned for three regions (i.e., temporal, central, and nasal)^(16)^.

Statistical analysis was performed with the StataCorp LP Stata 13 software to observe and compare both the groups. Qui-square test was applied for uniformity between the groups, while Fisher’s test was performed for parameters where the expected values were <5. The Shappiro-Wilk test was employed to verify the distribution on groups. Student’s t-test was applied for parametric parameters and Kruskal-Wallis test for applied for non-parametric parameters. A significance level of 5% (p<0.05) was accordingly adopted.

## RESULTS

For the CLDEQ-8 questionnaire translation and validation, a Portuguese version was applied twice by two different researchers on different occasions in a random sample of 30 subjects. The results obtained are summarized in table 1. Kappa agreement values were ≥0.7 in all questions, implying a good agreement between the two versions (English and Portuguese languages).

**Table 1:** Results of the CLDEQ-8 Portuguese questionnaire validation

Question	Kappa value		p-value
**1a**	0.70		<0.001
**1b**	0.70		<0.001
**2a**	0.74		<0.001
**2b**	0.71		<0.001
**3a**	0.72		<0.001
**3b**	0.72		<0.001
**4**	0.71		<0.001
**5**	0.70		<0.001

Hereafter, 61 subjects were enrolled in Group A and 20 in Group B. Table 2 displays the detailed features of each group.

**Table 2:** Demographics, ambient factors, and soft contact lens (SCL) wearing schedule in our study

		Group A (n=61)	Group B (n=20)	p-value
Age (years-old)		34 (19-60)	32 (24-38)	0.53^d^
Sex male/female (%)		26/74	40/60	0.05^l^
Air conditioner hours/day		5.1 (0-10)	4.7 (1-8)	0.34^d^
Computer display use hours/day		4.7 (0-10)	3.1 (1-8)	0.06^d^
Duration of SCL wear (years)		13.1 (1-43)	-	
SCL wear days/week		5.9 (2-7)	-	
SCL wear hours/day		12.4 (2-24)	-	

Data expressed in mean (minimum-maximum); SCL= soft contact lens; d= Mann-Whitney U-test; l= Fischer’s Exact test.

**Figure 2:**
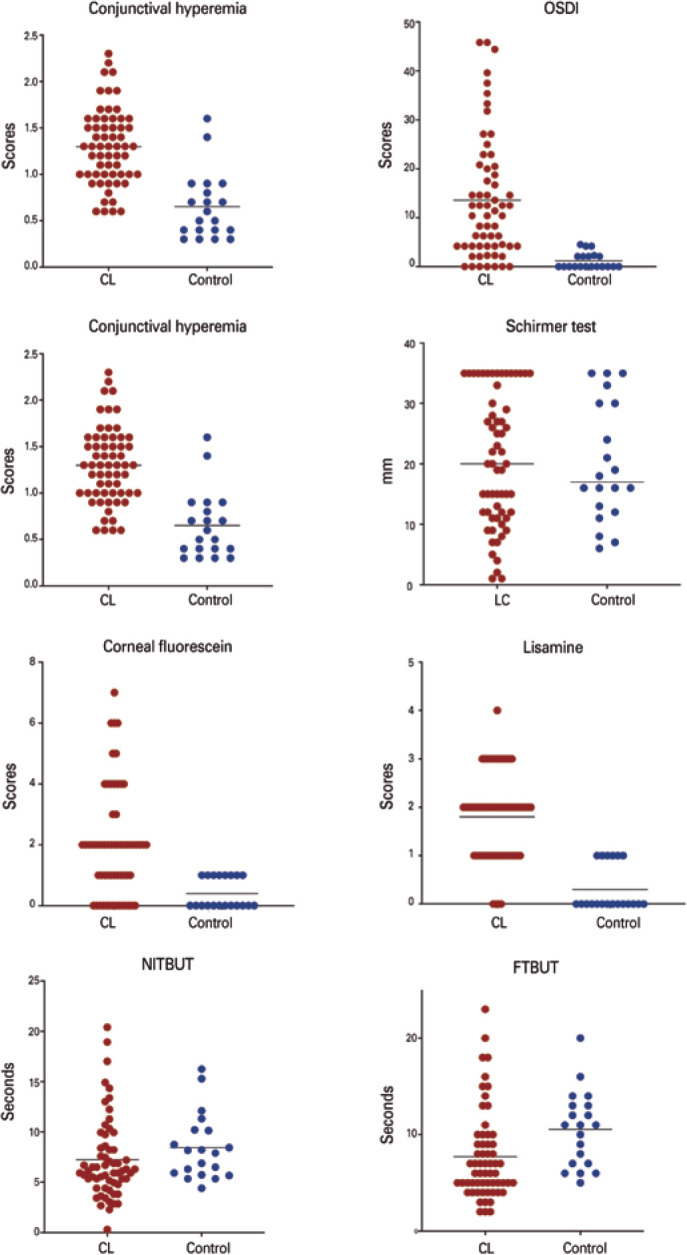
Comparisons of the main ocular surface parameters between contact lens wearers and the control subjects.

Notably, 47.5% (n=29) participants reported that they had to stop wearing contact lens for a limited time owing to discomfort from wearing. The CLDEQ-8 questionnaire results were 13.26 (1-28), which represented an intense or frequent dry eye symptom requiring therapy. Indeed, higher symptoms in OSDI were noted when compared to the controls. A comprehensive evaluation of the ocular surface parameters demonstrated a consisted impact of SCL on the ocular surface. Table 3 displays all objective and subjective parameters. Figure 2 indicates the scatter plots of each parameters and comparisons between the groups. All parameters differed in SCL wearers when compared to the controls, except in those related to tear volume, such as the TMH and Schirmer test.

**Table 3:** Ocular surface evaluation

	Group A	Group B	p-value
TMH	0.29 ± 0.16	0.25 ± 0.07	0.3833^d^
	(95% CI 0.2-0.3)	(95% CI 0.2-0.3)	
CH	1.29 ± 0.4	0.65 ± 0.36	0.0001^d^
	(95% CI 1.1-1.4)	(95% CI 0.4-0.8)	
Bulbar temporal CH	1.49 ± 0.53	0.62 ± 0.39	0.0001^d^
	(95% CI 1.3-1.8)	(95% CI 0.4-0.8)	
Bulbar nasal CH	1.44 ± 0.55	0.62 ± 0.32	0.0001^d^
	(95% CI 1.3-1.5)	(95% CI 0.4-0.7)	
Limbal temporal CH	0.91 ± 0.43	0.47 ± 0.24	0.0001^d^
	(95% CI 0.7-1.0)	(95% CI 0.3-0.5)	
Limbal nasal CH	0.93 ± 0.39	0.43 ± 0.18	0.0001^d^
	(95% CI 0.8-1.0)	(95% CI 0.3-0.5)	
NITBUT	7.24 ± 4.02	8.44 ± 3.27	0.0032^d^
	(95% CI 6.2-8.2)	(95% CI 6.9-9.9)	
Meiboscore inferior			<0.0001^l^
0	10 (16.39%)	8 (40%)	
1	34 (55.73%)	11 (55%)	
2	12 (19.67%)	1 (5%)	
3	3 (4.91%)	0 (0%)	
4	2 (3.27%)	0 (0%)	
Meiboscore superior			<0.0001^l^
0	0 (0%)	3 (15%)	
1	37 (60.65%)	16 (80%)	
2	17 (27.86%)	1 (5%)	
3	6 (9.83%)	0 (0%)	
4	1 (1.63%)	0 (0%)	
TBUT	7.70 ± 4.63	10.55 ± 3.88	0.0023^d^
	(95% CI 6.5-8.8)	(95% CI 8.7-12.3)	
FCS	2.0 ± 1.77	0.40 ± 0.50	0.0001^d^
	(95% CI 1.5-2.4)	(95% CI 0.1-0.6)	
Schirmer test	20.72 ± 10.9	20.05 ± 9.82	0.8086^d^
	(95% CI 17.9-23.5)	(95% CI 15.4-24.6)	
LOGSS	1.80 ± 0.89	0.30 ± 0.47	0.0001^d^
	(95% CI 1.0-2.6)	(95% CI 0.1-0.6)	
OSDI	13.58 ± 12.52	1.18 ± 1.63	0.0001^d^
	(95% CI 10.38-16.8)	(95% CI 0.4-1.9	

Data expressed in mean ± standard deviation; d= Mann-Whitney U-test; l= Fischer’s Exact test; OSDI= Ocular Surface Disease Index; TMH= tear meniscus height; CH= conjunctival hyperemia; NITBUT= non-invasive tear break-up time; TBUT= tear break-up time; FCS= fluorescein corneal staining; LGOSS= lissamine green ocular surface staining.

Scatter plots for comparisons of ocular surface parameters between contact lens wearers and control subjects. Data expressed in mean. OSDI= Ocular Surface Disease Index; NITBUT= non-invasive tear break-up time; TBUT= tear break-up time.

The sample size number of 20 in a proportion of 2:1 was planned, but as a greater number of contact lens wearers was enrolled and considered in the statistical analysis. To validate the power of our sample, we performed a *post hoc* analysis for each tested variable. This additional analysis reached higher powers for all statistically significant variables (0.88 a 1.0).

## DISCUSSION

This study revealed that a majority of SCL wearers were young females, in conformance to past studies, and 83.6% of all our subjects wore SCL for >8 h/day, which also agrees to the literature reports.

Chalmers and Begley^(19)^ reported an agreement between young age and dry eye symptoms among CL wearers. There were statistical differences in computers display exposure time between the groups, but it was not correlated with the frequency and intensity of the symptoms; this finding was consistent to those of other past studies^(19)^.

Punctate superficial keratitis is extremely common among contact lens wearers; however, it is a usual finding among the normal non-wearers. In the present study, corneal staining with fluorescein or lissamine green staining, respectively, on the cornea or ocular surface was higher among contact lens wearers. This finding is similar to that reported in the literature^(5,6,9,20,21)^.

Meibomian gland dysfunction and evaporative dry eye have been considered as the most prevalent form of dry eye signs across the world. A detailed evaluation through meibography examination and non-invasive and fluorescein TBUT were evaluated in this study, which confirmed that contact lens may profoundly interfere in these parameters, resulting in discomfort and discontinuation of contact lens wear^(7)^. Such findings corroborate with other reports in the literature^(20-23)^. Regarding the tear volume, no differences were noted in the present study patients^(7,20,24)^.

A detailed and objective measurements of conjunctival hyperemia may thus be considered as the hallmark of ocular surface inflammation^(20,21,25)^.

The OSDI is a well-known questionnaire developed in English^(15)^ and previously translated and validated to the Portuguese language^(18)^. It is widely used to measure the frequency of symptoms, environmental triggers, and vision related to the quality of life. In this study, the OSDI scores were greater amogn the SCL wearers, reflecting more symptoms and impact in their daily activities. Several past studies have confirmed a relationship between contact lens wear and dry eye symptoms^(4,7,26)^.

The CLDEQ-8 questionnaire was developed in English, published in 2012, and it was composed of eight questions in relation to the evaluation of the presence and magnitude of discomfort related to contact lens wear^(10,11)^. These studies highlighted, in the Tear Film and Ocular Surface Society consensus, that contact lens discomfort is an important diagnostic tool. One of the goals of the present study was to provide translation and validation of this questionnaire in Portuguese language, while following the standard procedures that are already well-defined in the literature^(12,13,27)^. Following this step, an important tool to screen and follow-up contact lens wearers is now available for incorporation in the clinical practices and researches in countries where Portuguese is the native language.

Our study recorded found CLDEQ-8 high scores in the SCL wearer group, indicating the magnitude of dry eye symptom among these subjects and the need for diagnostic evaluation and therapeutic approach. Chalmers^(11)^ stablished that a score of ≥12 in the CLDEQ-8 questionnaire represent an intense or frequent dry eye symptoms that requires therapy. This information is relevant considering that several studies have reported that discomfort and dry eye symptoms are the most common causes of discontinuing the use of contact lens^(28,29)^. In this study, we noted that 47.5% of the subjects interrupted contact lens wearing at some point of time out of discomfort and development of dry eye symptoms or other complications (such as infectious keratitis).

This study aimed to describe the impact of SCLs on the ocular surface and the magnitude of the related symptoms. In addition, the validated Portuguese version of the CLDEQ-8 questionnaire served as an important diagnostic tool for contact lens wearers. However, some limitations of this study need to be indicated, such as the small sample size, non-masking study, and the lack of detailed information about the types of products, lens materials, and multipurpose solutions used for cleaning and maintaining hygienic conditions. Some related risk factors to dry eye symptoms were however not evaluated, such as alcohol consumption and smoking.

This study provided a translated and validated Portuguese-language version of the CLDEQ-8 questionnaire, which represents an important tool for the evaluation of the experience of wearing contact lenses. The broad evaluation of the ocular surface performed in this study indicated an association between SCL wearing and ocular surface disturbances, suggesting the importance of diagnostic tests and the need for undertaking a therapeutic approach for dry eye symptoms amogn the SCL wearers.

## Supplementary files

**File 1:** CLDEQ-8 Portuguese version

Contact Lens Questionnaire-8 (CLDEQ-8)
Questions about **EYE DISCOMFORT:**
a. During a typical day in the past 2 weeks, **how often** did your eyes feel discomfort when wearing your contact lenses?
0 Never
1 Rarely
2 Sometimes
3 Frequently
4 Constantly
When your eyes felt discomfort with your contact lenses, **how intense was this feeling of discomfort?**
b. At the end of your wearing time?
Never Not at all Very
Have it Intense Intense
0___1 2___3 4___5
Questions about **EYE DRYNESS:**
a. During a typical day in the past 2 weeks, **how often** did your eyes feel dry?
0 Never
1 Rarely
2 Sometimes
3 Frequently
4 Constantly
When your eyes felt dry, **how intense was this feeling of dryness?**
b. At the end of your wearing time?
Never Not at all Very
Have it Intense Intense
0___1 2___3 4___5
Questions about **CHANGEABLE, BLURRY VISION:**
a. During a typical day in the past 2 weeks, **how often** did your vision change between clear and blurry or foggy when wearing your contact lenses?
0 Never
1 Rarely
2 Sometimes
3 Frequently
4 Constantly
When your vision was blurry, **how noticeable was the changeable, blurry, or foggy vision?**
b. At the end of your wearing time?
Never Not at all Very
Have it Intense Intense
0___1 2___3 4___5
**Contact Lens Questionnaire-8 (CLDEQ-8)**
1 Question about **CLOSING YOUR EYES:**
During a typical day in the past 2 weeks, **how often** did your eyes **bother you so much that you wanted to close them**?
0 Never
1 Rarely
2 Sometimes
3 Frequently
4 Constantly
Question about **REMOVING YOUR LENSES:**
How often during the past 2 weeks, did your eyes *bother you so much* while wearing your contact lenses that you felt as if you needed to stop whatever you were doing and **take out your contact lenses**?
1 Never
2 Less than once a week
3 Weekly
4 Several times a week
5 Daily
6 Several times a day

**File 2:** CLDEQ-8 English version

Questionário de Olho Seco em Usuário de Lente de Contato
**1. Questões sobre desconforto ocular**
c. Durante um dia comum, nas ultimas 2 semanas, com que frequência você sentiu desconforto ocular relacionado ao uso de suas lentes de contato?
0 Nunca
1 Raramente
2 Às vezes
3 Frequentemente
4 Constantemente
d. Na ocasião em que você sentiu desconforto com suas lentes de contato, qual intensidade ao final do período de uso?
Nunca senti Pouco Intenso Muito Intenso
0___1 2___3 4___5
**2. Questões sobre olho seco**
c. Durante um dia comum, nas ultimas 2 semanas, com que frequência você teve sensação de olho seco?
0 Nunca
1 Raramente
2 Às vezes
3 Frequentemente
4 Constantemente
d. Na ocasião em que você teve sensação de olho seco, qual intensidade ao final do período de uso das lentes de contato?
Nunca senti Pouco Intenso Muito Intenso
0___1 2___3 4___5
**3. Questões sobre embaçamento visual**
c. Durante um dia comum, nas ultimas 2 semanas, com que frequência você sentiu embaçamento visual durante o uso de suas lentes de contato?
0 Nunca
1 Raramente
2 Às vezes
3 Frequentemente
4 Constantemente
d. Na ocasião em que você sentiu embaçamento visual com suas lentes de contato, qual a intensidade deste sintoma ao final do período de uso?
Nunca senti Pouco Intenso Muito Intenso
0___1 2___3 4___5
**Questionário de Olho Seco em Usuário de Lente de Contato**
**4. Questão sobre fechamento ocular**
Durante um dia comum, nas últimas 2 semanas, com que frequência você sentiu tamanho desconforto ocular ao ponto de necessitar fechar os olhos?
0 Nunca
1 Raramente
2 Às vezes
3 Frequentemente
4 Constantemente
**5. Questão sobre remoção das lentes de contato**
Com que frequência nas últimas 2 semanas você sentiu tamanho desconforto ocular ao ponto de interromper alguma atividade e retirar as lentes de contato?
1 Nunca
2 Menos de uma vez por semana
3 Toda semana
4 Várias vezes durante a semana
5 Diariamente
6 Várias vezes ao dia
